# TLR4 Deficiency Protects against Hepatic Fibrosis and Diethylnitrosamine-Induced Pre-Carcinogenic Liver Injury in Fibrotic Liver

**DOI:** 10.1371/journal.pone.0158819

**Published:** 2016-07-08

**Authors:** Susanne Nicole Weber, Annika Bohner, Dianne H. Dapito, Robert F. Schwabe, Frank Lammert

**Affiliations:** 1 Department of Medicine II, Saarland University Medical Center, Homburg, Germany; 2 Department of Medicine, Columbia University, New York, NY, United States of America; University of Navarra School of Medicine and Center for Applied Medical Research (CIMA), SPAIN

## Abstract

**Background:**

The development of hepatocellular carcinoma (HCC) is a common consequence of advanced liver fibrosis but the interactions between fibrogenesis and carcinogenesis are still poorly understood. Recently it has been shown that HCC promotion depends on Toll-like receptor (TLR) 4. Pre-cancerogenous events can be modelled in mice by the administration of a single dose of diethylnitrosamine (DEN), with HCC formation depending amongst others on interleukin (IL) 6 production. Mice lacking the hepatocanalicular phosphatidylcholine transporter ABCB4 develop liver fibrosis spontaneously, resemble patients with sclerosing cholangitis due to mutations of the orthologous human gene, and represent a valid model to study tumour formation in pre-injured cholestatic liver. The aim of this study was to investigate DEN-induced liver injury in TLR4-deficient mice with biliary fibrosis.

**Methods:**

ABCB4-deficient mice on the FVB/NJ genetic background were crossed to two distinct genetic backgrounds (TLR4-sufficient C3H/HeN and TLR4-deficient C3H/HeJ) for more than 10 generations. The two congenic knockout and the two corresponding wild-type mouse lines were treated with a single dose of DEN for 48 hours. Phenotypic differences were assessed by measuring hepatic collagen contents, inflammatory markers (ALT, CRP, IL6) as well as hepatic apoptosis (TUNEL) and proliferation (Ki67) rates.

**Results:**

Hepatic collagen accumulation is significantly reduced in ABCB4^-/-^:TLR4^-/-^double-deficient mice. After DEN challenge, apoptosis, proliferation and inflammatory markers are decreased in TLR4-deficient in comparison to TLR4-sufficient mice. When combining ABCB4 and TLR4 deficiency with DEN treatment, hepatic IL6 expression and proliferation rates are lowest in fibrotic livers from the double-deficient line. Consistent with these effects, selective digestive tract decontamination in ABCB4^-/-^ mice also led to reduced tumor size and number after DEN.

**Conclusion:**

This study demonstrates that liver injury upon DEN challenge depends on pre-existing fibrosis and genetic background. The generation of ABCB4^-/^: TLR4^-/-^ double-deficient mice illustrates that TLR4-deficiency protects against hepatic injury in a preclinical mouse model of chronic liver disease.

## Introduction

Hepatic fibrosis represents a wound healing response to chronic liver damage and may lead to cirrhosis and hepatocellular carcinoma (HCC) which is the sixth most common cancer and the third cause of cancer-related deaths worldwide [1]. Liver fibrosis is commonly caused by environmental factors such as viral infections or malnutrition but the fact that fibrosis and its consequences differ between individuals with similar risk profiles [2] and the results of genome-wide association studies [3,4] point to a genetic predisposition, most likely mediated by multiple genes and their interaction with environmental factors.

Toll-like receptors (TLRs) belong to a large family of transmembrane proteins that recognize pathogen-associated molecular patterns (PAMPs) and are involved in innate immune responses [5,6]. In addition, they contribute to adaptive immune reactions and the regulation of sterile inflammation and tissue regeneration as well as carcinogenesis [7–9]. TLRs also play an important role in liver pathophysiology and chronic liver diseases, since the liver is constantly exposed to a high amount of PAMPs [10–12]. Especially TLR4, responsible for detecting lipopolysaccharide (LPS) from Gram-negative bacteria, has been shown to be expressed by nearly all cell types of the liver including hepatocytes [13], Kupffer cells [14], stellate cells [15], sinusoidal endothelial cells [16] and biliary epithelial cells [17]. In alcohol-induced liver injury, the activation of Kupffer cells depends on TLR4 [18], and in non-alcoholic fatty liver disease TLR4 deficiency reduces hepatic lipid accumulation and inflammation after a methionine-choline-deficient diet [19]. Hepatic fibrosis and cirrhosis have been linked to TLR4, with TLR4-deficient mice displaying less fibrosis in chemically (carbon tetrachloride, CCl_4_ or thioacetamide, TAA) or bile duct ligation (BDL)-induced fibrosis models [20]. In addition, TLR4-dependent participation in HCC has been demonstrated in mice, linking the promotion of liver carcinogenesis to intestinal microbiota [21]. Of note, in humans, a single nucleotide variation in the *TLR4* gene is associated with protection against fibrosis progression [22,23].

All these studies have been performed in either TLR4 mutant or deficient mice, or in the setting of chemical (CCl_4_) and surgical models (BDL). Although CCl_4_ and BDL are accepted models for the induction of fibrosis in mice and rats [2], they do not perfectly resemble the situation in patients with progressive liver fibrosis. In normal liver, the hepatocanalicular transporter ABCB4 (ATP-binding cassette transporter B4; a.k.a. MDR2 in mice and MDR3 in humans) translocates phosphatidylcholine from the hepatocyte into bile [24]. Mice that lack ABCB4 develop biliary fibrosis spontaneously [25]. Disruption of the transporter leads to toxic bile and cholangio- and hepatocellular damage, resulting in progressive intrahepatic cholestasis and sclerosing cholangitis [25]. First abnormalities in the liver are detected 3 weeks after birth and the fibrotic phenotype appears in the whole liver after 12 weeks [26]. On certain genetic backgrounds (129/Ola, FVB/N, BALB/c, C57BL/6), HCC formation has been reported between 6 and 18 months of age [26–28]. Differences in genetic backgrounds affect chronic hepatitis and HCC formation. For example, ABCB4-deficient mice on the C57BL/6 genetic background show less hepatic inflammation and HCC formation than on the FVB/N background [29].

In the present study, we combined the two genetic mouse models to establish a novel preclinical model for the analysis of TLR4 effects on liver fibrosis. ABCB4-deficient mice were crossed into the TLR4-deficient C3H/HeJ and the corresponding TLR4-sufficient C3H/HeN strains to obtain double-deficient and control lines, respectively. As first described by Poltorak and co-workers, C3H/HeJ mice carry a mutated form of the *Tlr4* gene (p.H712P) that results in TLR4 deficiency and defective LPS signaling [30]. Therefore they have widely been used and represent a valid model to study the effects of TLR4 deficiency throughout the organism [30]. As we also aimed to investigate the role of TLR4 with respect to very early cancerogenous events we furthermore combined the ABCB4/TLR4-deficiency model with diethylnitrosamine (DEN) treatment to analyse short-term DEN responses in fibrotic liver.

## Methods

### Mice

Mice were hosted in individually ventilated cages under standard conditions (12L:12D photoperiod) and received water and a standard rodent diet *ad libitum*. The animals were monitored daily, and special care was taken to observe any signs of abnormalities (i.e. marked weight loss or altered behaviour), which did not appear.

This study was carried out in strict accordance with all relevant welfare regulations and the Animal Care and Use Committee for Saarland University. The protocol was approved by the Committee on the Ethics of Animal Experiments of the Landesamt für Verbraucherschutz, Saarbrücken, Saarland; Permit Number: TV22/2008 and TV36/2011). To generate ABCB4^-/-^:TLR4^+/+^ and ABCB4^-/-^:TLR4^-/-^ mouse lines respectively, ABCB4-deficient mice on the FVB/NJ background (strain #002539, The Jackson Laboratory) were crossed to strains C3H/HeN (TLR4-susceptible, Charles River) and C3H/HeJ (TLR4-deficient, strain #000659, The Jackson Laboratory) for more than 10 generations. All animals developed normal, and no mortality or illness was observed. Genotyping of the *Abcb4* allele was carried out as described [31]. The mutated *Tlr4* allele of C3H/HeJ was detected by allelic discrimination using a SNP assay for *rs3023006* (Life Technologies). SNP genotyping was carried out on the Taqman® 7500 Fast Real-Time PCR System (Life Technologies).

For modelling short-term DEN responses, individual mice (n = 12 per line; 6 males, 6 females) were injected intraperitoneally with a single dose of diethylnitrosamine (DEN; 100 mg/kg body weight, Sigma-Aldrich) [32]. After 48 hours, mice were sacrificed and compared to untreated controls (n = 6 per line; 3 males, 3 females). Again, all mice behaved normal and did not show any signs of distress upon DEN administration. For sacrifice, mice were submitted to an overdose of isoflurane.

To study of the effects of gut sterilization on HCC development, mice (n = 6) received a combination of ampicillin (1 g/l), neomycin (1 g/l), metronidazole (1 g/l) and vancomycin (500 mg/l) in drinking water after DEN challenge (at the age of 2 weeks) for 24 weeks as described [21].

### Chemical assays

For determination of plasma alanine aminotransferase (ALT) activities, blood was taken prior to DEN injection and after sacrifice. After centrifugation, plasma ALT activities were measured with an Olympus AU400 chemistry analyzer, using adapted reagents provided by Olympus [31].

The quantification of hepatic collagen contents was performed in liver hydrolysates via photometric measurement of the collagen-specific amino acid hydroxyproline as described [33,34].

### Immunohistochemistry

Apoptotic cells were analysed using the Apoptag® Peroxidase In Situ Apoptosis Detection Kit (TUNEL method; Millipore). In brief, paraffin slides were deparaffinised in a descending alcohol series, treated with proteinase K and H_2_O_2_, and incubated with Tdt enzyme at 37°C for one hour. Afterwards, slides were washed and treated with peroxidase. Apoptotic cells were visualized with AEC (DAKO), counter stained with haematoxylin, and covered with AquaTex® (Merck).

For proliferation analyses, paraffin sections were deparaffinised, treated with H_2_O_2_ and citrate buffer (0.1M, microwave, 25 min), and incubated with a monoclonal rat Ki67 antibody (4°C o/n, 1:100; DAKO). Secondary antibody (biotinylated-rabbit-anti-rat, 1:100; DAKO) and ABC complex (Vectastain) were applied thereafter, and Ki67 positive cells were visualized in the same way as TUNEL positive cells.

For each assay, two slides per animal were stained, and five representative high power fields (HPFs; 40× magnification) per section were evaluated using LAS Software (Leica DM5000B). Positively stained cells were counted, and the relative frequency was determined as the mean in five HPFs.

### Gene expression analyses

RNA from snap-frozen livers was isolated using RNeasy Mini Kit (Qiagen). One μg of RNA was transcribed to cDNA using the High Capacity cDNA Reverse Transcription Kit (Life Technologies). Expression of Collagen 1α1 (*Col1a1*; Mm00801666_g1), α-Smooth muscle actin *(a-SMA*; Mm_00725412_s1), Interleukin 6 (*ll6*; Mm00446191_m1) and C-reactive protein (*Crp*; Mm00432680_g1) was carried out on the Taqman® 7500 Fast Real-Time PCR System. *Gapdh* (Mm99999915_g1) served as endogenous control. Results were evaluated using the ΔΔCT method and normalised to controls.

### Statistical analyses

Data are presented as means ± SEM. Statistical analyses were carried out using SPSS 20.0 and GraphPad Prism 5.03 software. One-way ANOVA or t-test, respectively, was performed and p-values < 0.05 were considered as statistically significant.

## Results

### TLR4 deficiency and biliary fibrosis

The effect of TLR4 on the development of hepatic fibrosis has so far been investigated in artificial models only with fibrosis induced by chemicals (CCl_4_, TAA) or surgery (BDL). To analyze the effect of TLR4 in a more representative model of human liver fibrosis, ABCB4-deficient mice were crossed into the TLR4-deficient background to obtain double-deficient animals. The resulting ABCB4^-/-^:TLR4^+/+^ and ABCB4^-/-^:TLR4^-/-^ lines were compared with respect to fibrosis severity.

**Fig 1:**
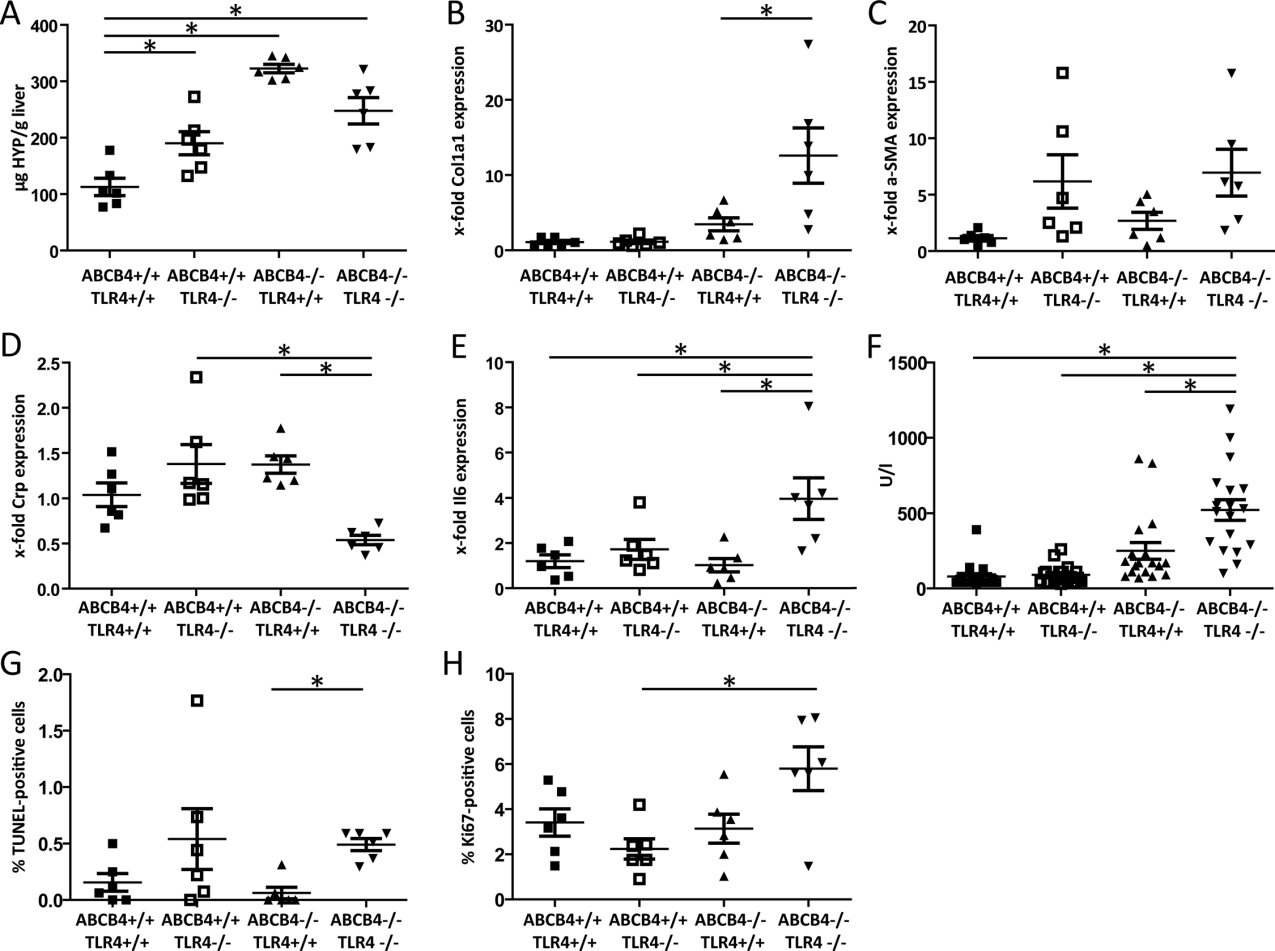
Fibrosis progression depends on TLR4 status. ABCB4-deficient, ABCB4/TLR4-double-deficient and the corresponding control mice were compared with respect to hepatic damage and fibrosis at 16 weeks of age when fibrosis is already established (n = 6 per line; 3 males, 3 females). (A) Hepatic collagen contents, measured as μg hydroxyproline (HYP) per g liver. (B) Relative hepatic *Col1a1* mRNA expression. ABCB4^+/+^:TLR4^+/+^ mice were set as 1. (C) Relative hepatic *a-Sma* mRNA expression. ABCB4^+/+^:TLR4^+/+^ mice were set as 1. (D) Relative hepatic *Crp* mRNA expression. ABCB4^+/+^:TLR4^+/+^ mice were set as 1. (E) Relative hepatic *Il6* mRNA expression. ABCB4^+/+^:TLR4^+/+^ mice were set as 1. (F) Plasma alanine aminotransferase (ALT) activities, measured in units per liter (U/l). (G) Hepatocellular apoptosis rates. (H) Hepatocellular proliferation rates. *p < 0.05.

Panel A of Fig 1 displays that ABCB4-deficient mice lacking TLR4 show significantly less hepatic collagen accumulation compared to ABCB4-deficient/TLR4-sufficient mice, as measured by the amounts of the collagen-specific amino acid hydroxyproline. The difference between strains ABCB4^+/+^:TLR4^+/+^ and ABCB4^-/-^:TLR4^+/+^ is more marked than between ABCB4^+/+^:TLR4^-/-^ and ABCB4^-/-^:TLR4^-/-^ mice, consistent with the modulation of disease severity by TLR4. *Col1a1* mRNA levels are most strongly elevated in the double-deficient mice (Fig 1B), pointing to differences in post-translational modification and/or collagen composition between mouse lines. There are no significant differences in a-*Sma* expression in the four strains, implicating only a mild effect of TLR4 on this parameter (Fig 1C). Whereas hepatic *Crp* mRNA levels are significantly lower (Fig 1D), *Il6* expression levels are elevated significantly, consistent with a higher IL6-dependent inflammatory response in the absence of TLR4 (Fig 1E). Plasma ALT activities are significantly higher in the double-deficient mice (Fig 1F). Also, apoptosis and proliferation rates are increased significantly (Fig 1G and 1H). Taken together, these findings indicate moderately reduced fibrosis but a higher inflammatory response in livers from TLR4-deficient ABCB4 knockout mice.

### TLR4 deficiency protects healthy liver against DEN-induced liver injury

**Fig 2:**
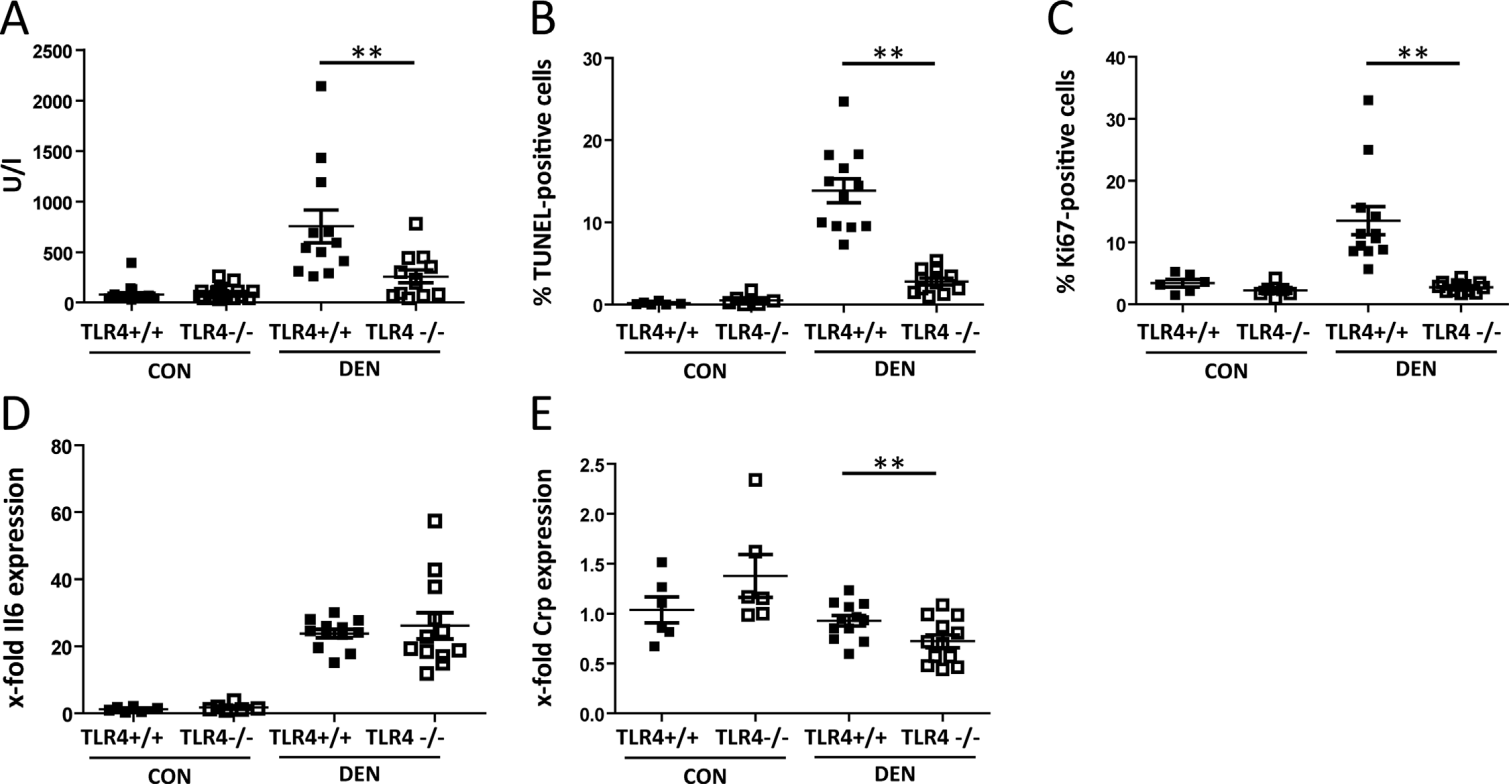
DEN-induced liver injury is reduced in the absence of TLR4. TLR4- sufficient and deficient animals at the age of 16 weeks were subjected to DEN for 48 hours (DEN, n = 12 per line; 6 males, 6 females) and compared to untreated controls (CON; n = 16per line; 3 males, 3 females). (A) Plasma ALT activities, measured in U/l. (B) Hepatocellular apoptosis rates. (C) Hepatocellular proliferation rates. (D) Relative hepatic *Il6* expression. Untreated TLR4-sufficient mice were set as 1. (E) Relative hepatic *Crp* expression. Untreated TLR4-sufficient mice were set as 1. **p<0.01.

To analyze the role of TLR4 in short-term DEN treatment in healthy liver, TLR4-sufficient C3H/HeN and TLR4-deficient C3H/HeJ mice were treated with a single dose of DEN for 48 hours at the age of 16 weeks and compared to untreated controls [32]. Upon DEN challenge, plasma ALT activity, as an indicator of acute liver injury, increases in both strains, with TLR4-sufficient mice showing significantly higher ALT levels in comparison to TLR4-deficient animals (Fig 2A). As a characteristic of acute liver injury, apoptosis and proliferation rates were determined. Apoptosis increases after DEN treatment in both strains, with the TLR4-deficient strain showing significantly less TUNEL-positive cells as compared to TLR4-sufficient mice, reflecting ameliorated liver damage in the absence of TLR4 (Fig 2B). Proliferation was equally affected, since TLR4-sufficient animals display significantly increased proliferation rates after DEN injection in comparison to TLR4-deficient mice (Fig 2C). Hepatic expression of *Il6*, as a marker for the inflammatory response, is augmented in DEN-treated mice, but there is no difference with respect to TLR4 status (Fig 2D). Although *Crp* expression is not significantly affected by DEN, TLR4-deficient mice show the lowest *Crp* levels (Fig 2E).

### TLR4 modulates the response to DEN in injured liver

**Fig 3:**
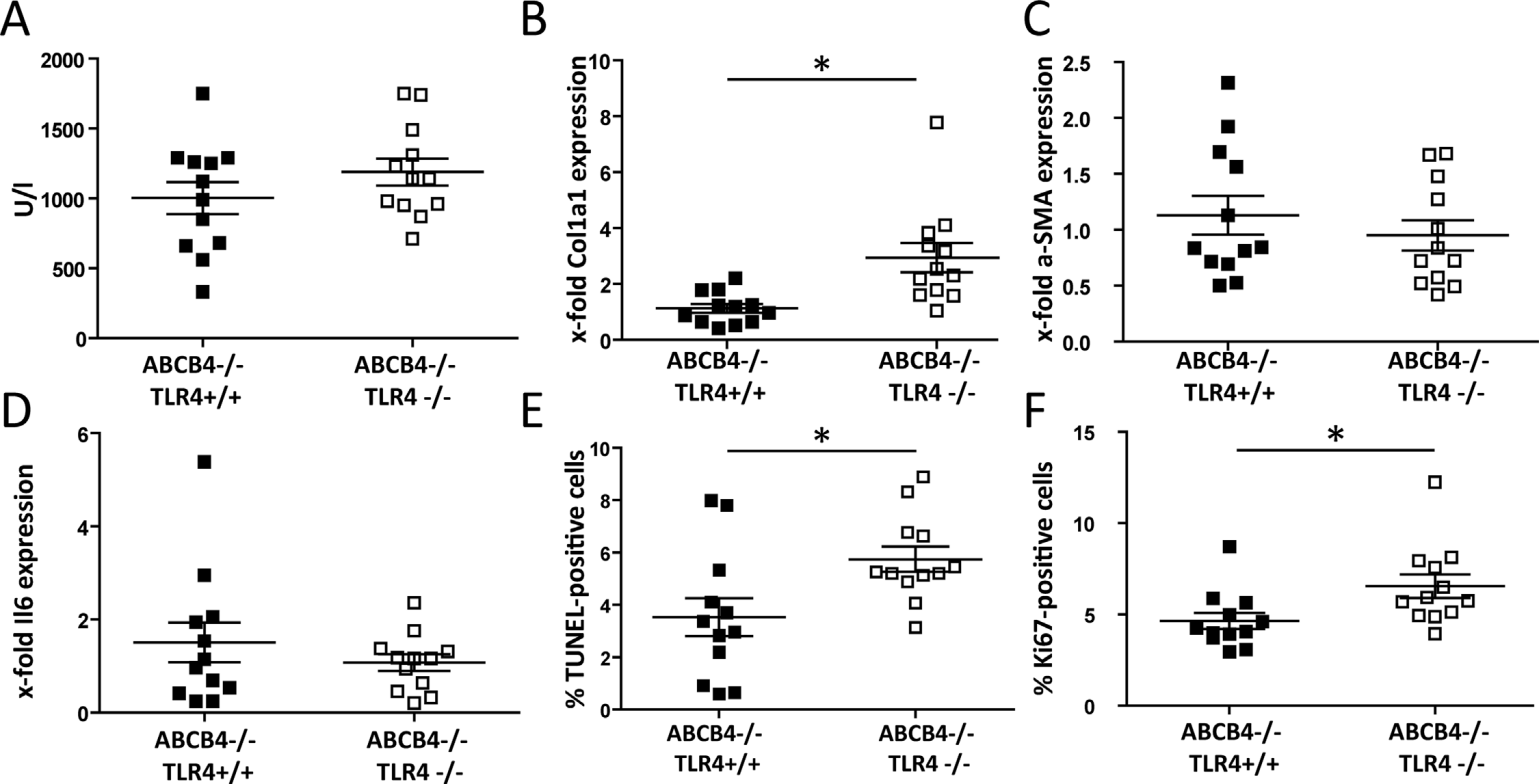
TLR4 deficiency increases apoptosis and proliferation at early fibrotic stages. ABCB4-deficient and ABCB4/TLR4-double-deficient mice were subjected to DEN at 7 weeks of age (n = 12 per line; 6 males, 6 females). (A) Plasma ALT activities, measured in U/l. (B) Relative hepatic *Col1a1* mRNA expression. ABCB4^-/-^:TLR4^+/+^ mice were set as 1. (C) Relative hepatic *a-SMA* mRNA expression. ABCB4^-/-^:TLR4^+/+^ mice were set as 1. (D) Relative hepatic *Il6* expression. ABCB4^-/-^:TLR4^+/+^ mice were set as 1. (E) Hepatocellular apoptosis rate. (F) Hepatocellular proliferation rate. *p < 0.05.

**Fig 4:**
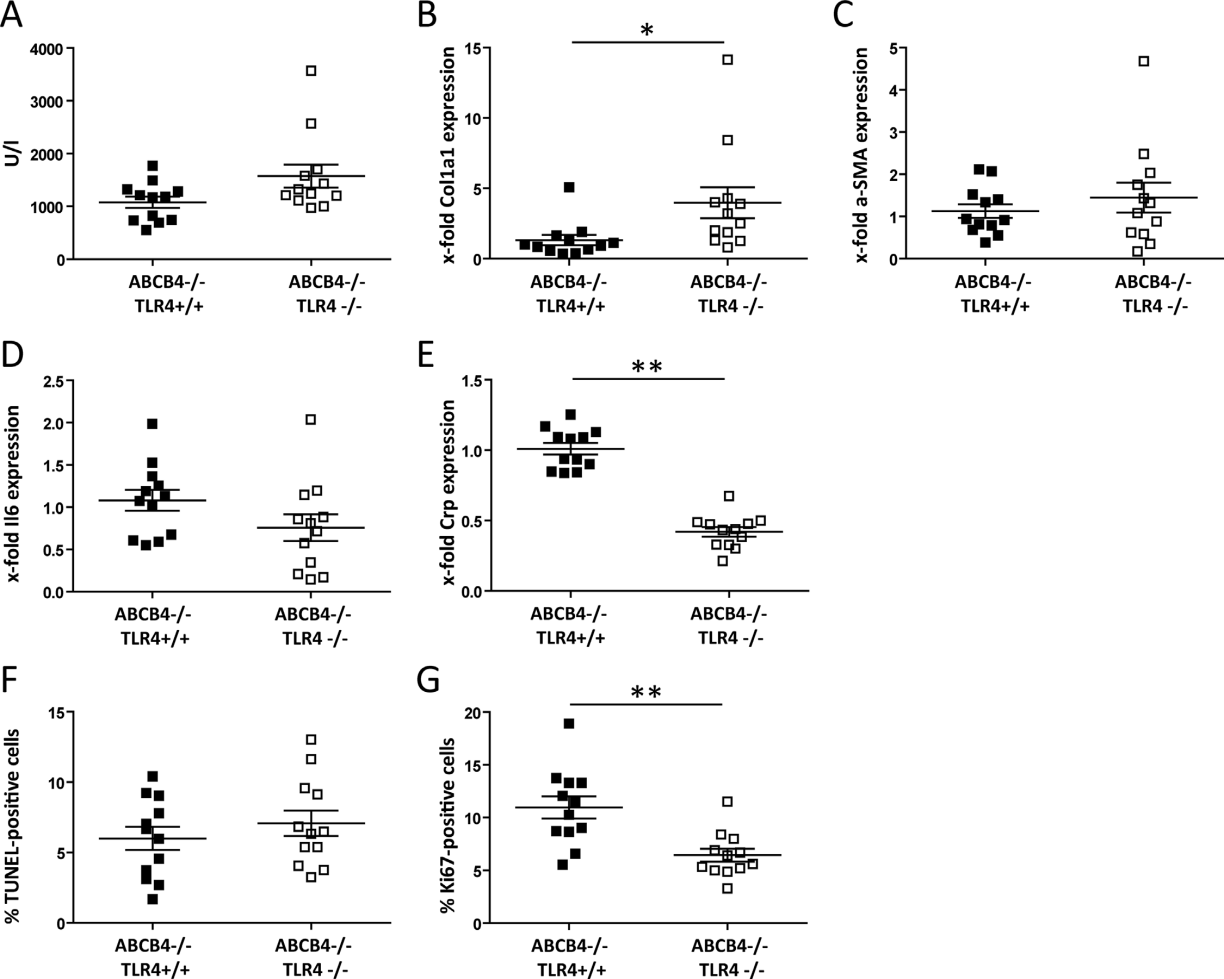
TLR4 deficiency protects against DEN-induced liver injury in fibrotic liver. ABCB4-deficient and ABCB4/TLR4-double-deficient mice were subjected to DEN at 16 weeks of age (n = 12 per line; 6 males, 6 females). (A) Plasma ALT activities, measured in U/l. (B) Relative hepatic *Col1a1* mRNA expression. ABCB4^-/-^:TLR4^+/+^ mice were set as 1. (C) Relative hepatic *a-SMA* mRNA expression. ABCB4^-/-^:TLR4^+/+^ mice were set as 1. (D) Relative hepatic *Il6* expression. ABCB4^-/-^: TLR4^+/+^ mice were set as 1. (E) Relative hepatic *Crp* expression. ABCB4^-/-^:TLR4^+/+^ mice were set as 1. (D) Hepatocellular apoptosis rate. (E) Hepatocellular proliferation rate. *p<0.05; **p<0.01.

As HCC formation usually occurs on a fibrotic background, we combined the two mouse models, ABCB4/TLR4-deficiency and short-term DEN treatment. TLR4-sufficient and TLR4-deficient ABCB4 knockout mice were treated with DEN at 7 weeks (pre-fibrotic stage; Fig 3) and 16 weeks of age (established fibrosis; Fig 4), as described in Methods. At the early time-point, liver damage (as assessed by ALT levels; Fig 3A) is not altered. *Col1a1* mRNA levels are significantly elevated in the double-deficient mice (Fig 3B), whereas *a-Sma* expression does not differ (Fig 3C). The inflammatory response (as indicated by hepatic *Il6* expression; Fig 3D) is not affected. However, panels E and F of Fig 3 illustrate that ABCB4/TLR4 double-deficient mice show higher apoptosis and proliferation rates, respectively, after DEN treatment in comparison to ABCB4 knockout mice.

In the congenic mouse lines with liver fibrosis at 16 weeks of age, ALT activities and apoptosis rates are not significantly changed (Fig 4A and 4F). *Col1a1* expression increases in the double-deficient mice (Fig 4B) while *a-Sma* mRNA levels remain constant (Fig 4C).

In contrast, *Il6* and *Crp* mRNA levels are lower as compared to ABCB4 deficient mice with normal TLR4 status (Fig 4D and 4E). Also, proliferation rates are significantly decreased in ABCB4/TLR4 double-deficient mice (Fig 4G).

### Selective digestive tract decontamination reduces tumor growth

**Fig 5:**
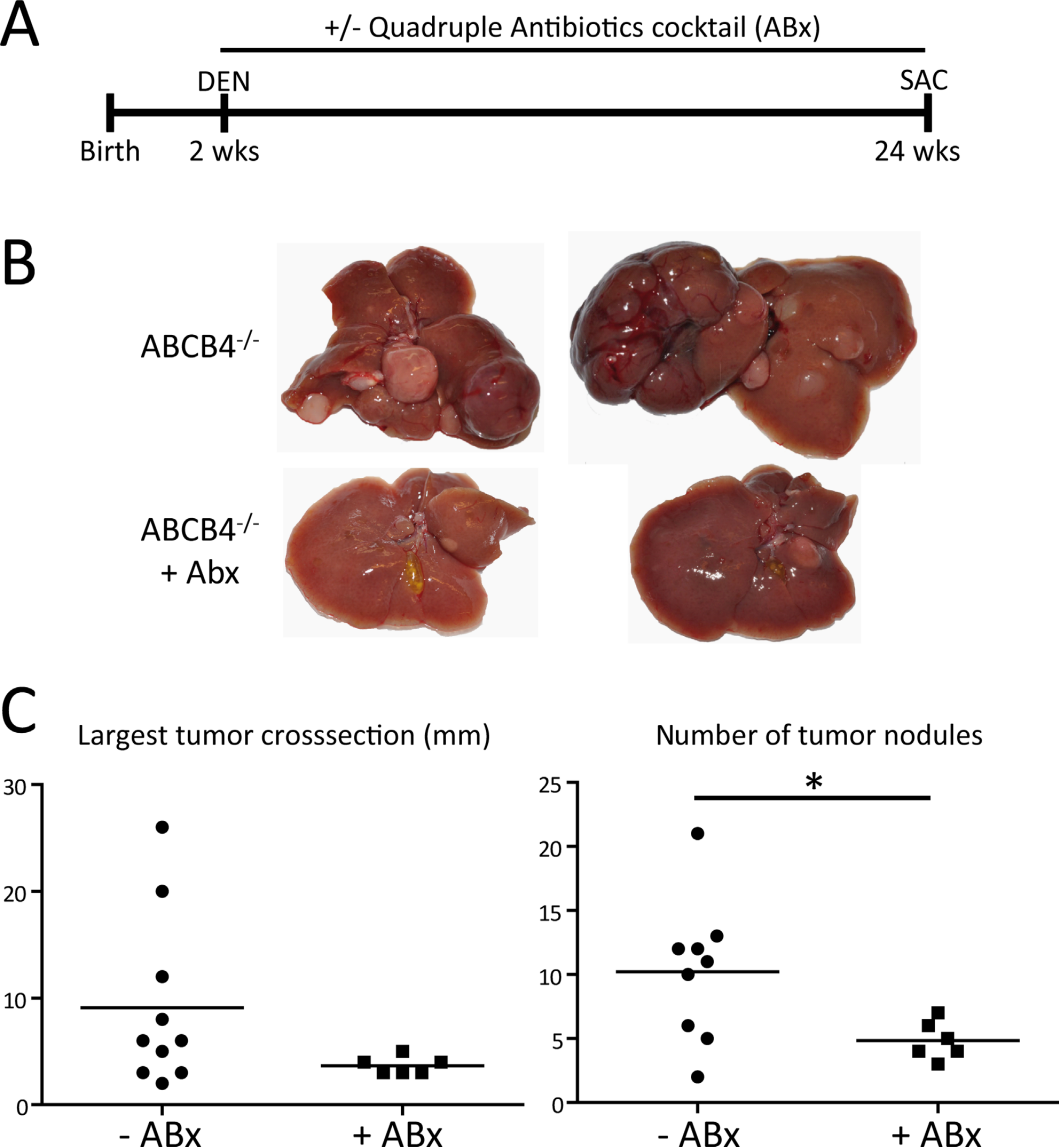
Gut sterilization leads to reduced tumor growth. ABCB4-deficient mice underwent DEN treatment and received a combination of four antibiotics (Abx) in drinking water until sacrifice (SAC). (A) Experimental design. (B) Macroscopic appearance of livers from ABCB4-deficient mice with or without Abx treatment. (C) Tumor sizes and numbers in the two experimental groups (-ABx: n = 10; +ABx: n = 6). *p<0.05.

To model the effects of TLR4 deficiency on HCC development, we sterilized the gut with a cocktail of antibiotics in ABCB4-deficient mice after DEN treatment (Fig 5A). The use of antibiotics lead to reduced tumor growth and significantly lower tumor numbers, indicating that HCC development is promoted by intestinal bacteria (Fig 5B and 5C).

## Discussion

As a modulator of innate immunity and LPS response, TLR4 has been widely implicated in various liver disorders including liver fibrosis and hepatocellular carcinoma [8,11]. Many studies linked TLR4 activity to enhanced fibrosis but all of these experiments were performed using chemical or surgical models [20]. In this study, we aimed to set up a preferable pathophysiological situation, which allows us to better assess the role of TLR4 in hepatic fibrosis and pre-cancerogenous liver injury. We took advantage of C3H/HeJ mice that carry a loss-of-function mutation in the *Tlr4* gene [30] and ABCB4 knockout mice that develop biliary fibrosis [28], and generated congenic ABCB4-deficient mice with and without TLR deficiency. ABCB4-deficient mice lacking TLR4 show increased liver damage, hepatic inflammatory response (*Il6* expression), apoptosis and proliferation as compared to ABCB4 knockout mice sufficient for TLR4. This imbalance in "liver tolerance" has been linked to TLR4 before [10,11]. Interestingly, although liver damage and cell division/death are more prominent in the double-deficient line, hepatic collagen contents as marker for liver fibrosis are reduced, implicating a beneficial role for TLR4 in liver injury but not in liver fibrosis in this model. This differential role of TLR4 might be due to interaction with the pre-existing liver disease (ABCB4 deficiency).

As established by Naugler et al. acute liver injury can be modeled by a single dose of DEN for 48 hours [32]. In this study the authors describe IL6-dependent gender-specific differences in response to DEN. Interestingly we only detected gender disparity in ALT activity (S1 Fig) in our different models, all other parameters measured showed no significant differences between male and female mice (data not shown).

The roles of TLR4 in liver cancer have been analyzed in several studies showing an inhibitory effect on HCC initiation in the DEN model [35] and HCC promotion in a combined model of CCl_4_ and DEN [21]. In our study we could show that in the absence of TLR4, liver damage, apoptosis and proliferation after DEN challenge are decreased, indicating that TLR4 promotes DEN-induced liver injury in mice and that the inactivation of TLR4 is advantageous in this setting. Nevertheless, Wang et al. (36) showed a protective role of TLR4 in a long-term DEN model in TLR4-deficient and wild-type mice. These mice were injected shortly after birth without pre-damaged liver. This might explain our beneficial role of TLR4 in our combined ABCB4/TLR4-deficiency model as the carcinogenic stimulus is brought to an already injured liver.

In the third part of our study, mice lacking ABCB4 and TLR4 were subjected to DEN at two defined points in time (pre-fibrosis and established fibrosis) to assess the situation of hepatic injury in a fibrotic liver. At seven weeks of age, only minor alterations are present in livers of ABCB4-deficient mice [24,25]. In our model no differences with respect to liver damage and inflammatory response are apparent, while apoptosis and proliferation rates are increased in ABCB4^-/-^:TLR4^-/-^ double-deficient animals as compared to congenic ABCB4^-/-^:TLR4^+/+^ mice. This observation indicates that TLR4 modifies the hepatocellular response at this early stage and is consistent with the notion that TLR4 deficient mice display increased apoptosis in non-tumorous liver tissue [21].

Later at 16 weeks of age when biliary fibrosis is established in ABCB4 knockout mice, the additional ablation of TLR4 leads to lower proliferation rates, while apoptosis tends to be elevated in the short-term DEN model; as expected, inflammatory markers are also decreased. These changes should have beneficial effects on hepatocytes, as unlimited division capacity and reduced programmed cell death are hallmarks of cancer [36]. The results are also consistent with reduced expression of the antiapoptotic genes *Birc3*, *Birc5* and *Nos2* in livers from TLR4^-/-^ mice suggesting that TLR4 mediates survival ligands to tumor initiating cells [21].

Since TLR4 deficiency showed protective effects in our model, we additionally analyzed the effect of gut sterilization after HCC induction by DEN in ABCB4-deficient mice. This model resembled the findings in TLR4-deficient mice, with the reduction of HCC number in gut sterilized mice fitting to the observations in our genetically modified mice as well as other reports in experimental HCC models [35,37,38].

As C3H/HeJ and C3H/HeN inbred strains are kept separately since 1974 when C3H/HeN came to Europe we certainly cannot exclude that other genetic differences apart from *Tlr4* may contribute to our results but taken together, our study supports the hypothesis that TLR4 plays an important role in both hepatic fibrosis and DEN-induced liver injury. TLR4 deficiency exerts beneficial effects on liver fibrogenesis, as indicated by reduced hepatic collagen accumulation in ABCB4^-/-^:TLR4^-/-^ double-deficient mice. In addition, the absence of TLR4 has protective effects against pre-cancerogenous events in cholestatic liver injury, pointing to new avenues for the prevention and therapy of cancer in ABCB4 deficiency and related cholestatic liver diseases.

## Supporting Information

S1 Fig ALT activities divided by sex. Results of Figs 1F(A), 2A(B) and 4A(C) itemized by sex (m: male, f: female).(A) Plasma ALT activities, measured in U/l of ABCB4-deficient and ABCB4/TLR4-double-deficient mice. (B) Plasma ALT activities, measured in U/l of TLR4- sufficient and deficient animals at the age of 16 weeks with (DEN) and without (CON) DEN challenge. (C) Plasma ALT activities, measured in U/l of ABCB4-deficient and ABCB4/TLR4-double-deficient mice subjected to DEN at 16 weeks of age. *p<0.05.
(TIF)
