# Supplementary figures and images for: TLR4 Deficiency Protects against Hepatic Fibrosis and Diethylnitrosamine-Induced Pre-Carcinogenic Liver Injury in Fibrotic Liver
Source: PLoS One. 2016 Jul 8;11(7):e0158819. doi: 10.1371/journal.pone.0158819 (PMC4938399; doi:10.1371/journal.pone.0158819)

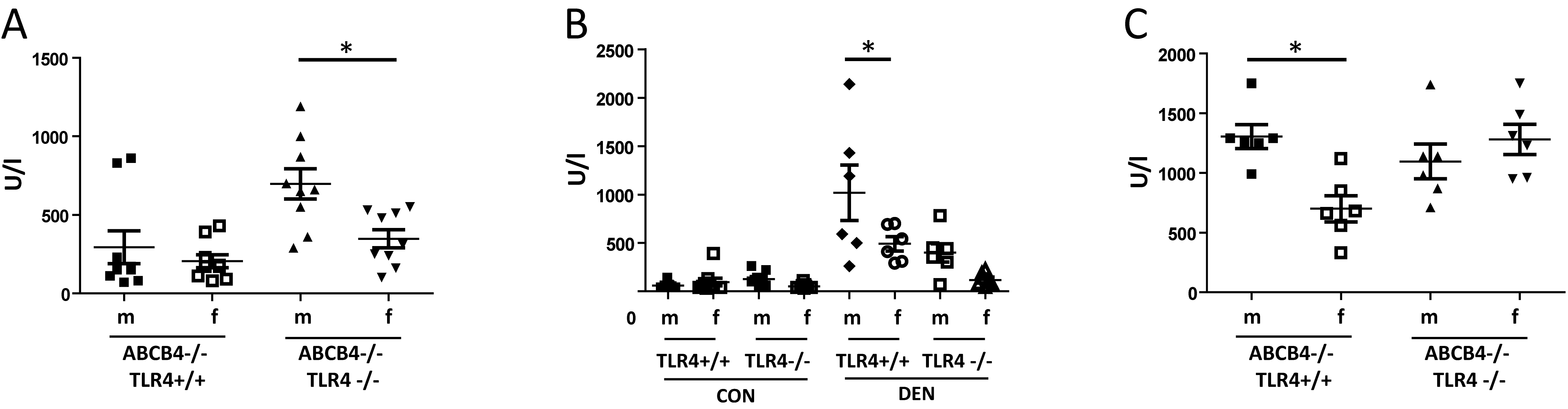

Supplement: S1 Fig — Results of Figs 1F(A), 2A(B) and 4A(C) itemized by sex (m: male, f: female).(A) Plasma ALT activities, measured in U/l of ABCB4-deficient and ABCB4/TLR4-double-deficient mice. (B) Plasma ALT activities, measured in U/l of TLR4- sufficient and deficient animals at the age of 16 weeks with (DEN) and without (CON) DEN challenge. (C) Plasma ALT activities, measured in U/l of ABCB4-deficient and ABCB4/TLR4-double-deficient mice subjected to DEN at 16 weeks of age. *p<0.05. (TIF) [file pone.0158819.s001.tif]
